# A Few-Channel Brain–Computer Interface System Based on a Heuristic Algorithm

**DOI:** 10.3390/biomimetics11090613

**Published:** 2026-09-01

**Authors:** Junhong Luo, Jianbin Yu, Hui Cao, Qiyue Tan, Jinheng Chen, Jing Xiao

**Affiliations:** 1 School of Artificial Intelligence, Guangzhou Maritime University, Guangzhou 510725, China; luojunhong1@mails.gdut.edu.cn (J.L.); yujianbin@e.gzhu.edu.cn (J.Y.); caohui@gzmtu.edu.cn (H.C.); tanqiyue@gzmtu.edu.cn (Q.T.); chenjinheng@gzmtu.edu.cn (J.C.); 2 School of Mathematics and Statistics, Guangdong University of Technology, Guangzhou 510520, China; 3 School of Electronics and Communication Engineering, Guangzhou University, Guangzhou 510006, China

**Keywords:** brain–computer interface (BCI), P300, channel selection, genetic algorithm, user experience

## Abstract

Traditional P300 brain–computer interface (BCI) systems rely on multi-channel EEG acquisition, causing cumbersome setup, lengthy preparation, and high user workloads, which limits their real-world application. To enhance practicality, this paper proposes a fixed few-channel selection framework based on a heuristic algorithm to balance decoding performance and user experience. We integrated a genetic algorithm (GA) with Bayesian linear discriminant analysis (BLDA) to identify a strongly generalizable few-channel combination from a traditional eight-channel system, avoiding costly subject-specific recalibration. Validating this method, 48 healthy subjects completed rigorous offline and online virtual reality (VR) experiments. Results showed that the proposed three-channel system maintained highly comparable accuracy and information transfer rates to the eight-channel system, showing no significant performance degradation. Crucially, the few-channel scheme reduced equipment preparation time by 90% (from 30 to 3 min). Furthermore, NASA-TLX workload evaluations confirmed a significant reduction in users’ psychological and physical burdens (*p* < 0.05). Ultimately, while preserving core interaction performance, this few-channel strategy vastly improves user experience and system practicality, offering key theoretical and practical support for implementing lightweight, user-friendly BCI systems.

## 1. Introduction

Brain–computer interfaces (BCIs), as a frontier technology in human–machine interaction [1], enable patients with motor impairments and spinal cord injuries to control external devices by decoding brain neural signals [2,3,4]. Particularly, P300-based BCI systems demonstrate compelling advantages in the operation of external devices such as home appliances, wheelchairs, and robots. These benefits stem fundamentally from their independence from extensive user training and their rapid response capabilities, executing command recognition typically within 2 to 5 s [5,6,7,8].

Traditional P300-BCI systems typically rely on 8- to 32-channel EEG acquisition devices. While these high-density setups capture comprehensive EEG signals, their inherent equipment complexity severely restricts system practicality. For instance, installing electrodes and applying conductive gel require over 30 min of preparation before an experiment, followed by mandatory scalp cleaning afterward. These cumbersome procedures greatly reduce patients’ willingness to use the system [9,10,11]. Furthermore, the high computational complexity associated with multi-channel data limits real-time interaction performance [12,13,14]. Although dry electrode technology shortens setup time [11], it often suffers from high impedance and noise, especially in areas with dense hair. This results in a lower signal-to-noise ratio compared to wet electrodes [15], and fails to resolve the fundamental issues of multi-channel systems.

To address this, researchers have proposed few-channel optimization strategies, which aim to improve system practicality by minimizing the number of electrodes without compromising decoding performance [16]. For example, Nijboer et al. [17] selected typical channels based on empirical findings by Krusienski et al. [9]. Alternatively, Fakhr et al. adopted a recursive algorithm to evaluate a support vector machine (SVM) classifier. By iteratively removing one channel at a time and discarding those whose removal improved classifier performance, they identified the optimal electrode subset.

While these methods effectively reduce the channel count, their inefficient selection schemes still consume significant preparation time. In recent years, metaheuristic algorithms—such as multi-objective particle swarm optimization (MOPSO) [13] and the non-dominated sorting genetic algorithm II (NSGA-II) [18]—have been utilized for automatic channel selection. However, these algorithms require repeated model training for each subject, resulting in poor cross-subject generalization [19]. Furthermore, the performance of existing few-channel schemes remains inferior to full-channel configurations. For instance, the accuracy of eight-channel systems in P300 spelling tasks is typically lower than that of 32-channel setups [13], making them inadequate for practical applications. Additionally, traditional BCI systems suffer from high training costs. Most systems require large amounts of calibration data (often hundreds of repeated stimuli) to train accurate classification models. This process is highly time-consuming—often taking one to two hours per subject and can degrade signal quality due to subject fatigue [12]. While some studies have attempted to reduce this burden using transfer learning or transfer classification [14], high inter-subject variability in EEG features severely limits their generalization capabilities. Consequently, achieving true “zero-training” or “few-training” brain–computer interaction remains a significant challenge. More recently, researchers have explored online transfer learning strategies, such as supervised self-training, to continuously fine-tune pre-trained models using online data, moving toward calibration-free BCI operation [20]. Furthermore, advancing the decoding efficiency and practical deployment of EEG-based interfaces continues to be a major focus in recent studies. For example, Gkintoni and Halkiopoulos systematically reviewed the mapping of EEG metrics to cognitive models to optimize analytical frameworks [21]. To enhance system practicality and user experience, Mounesi Rad and Danishvar proposed a novel dry electrode combined with deep convolutional graph networks for accurate emotion recognition [22]. Additionally, in the context of clinical applications such as ADHD detection, Attallah demonstrated the importance of optimal channel selection and multi-resolution analysis to reduce computational complexity and improve diagnostic accuracy [23]. These continuous advancements highlight a collective trend in the field toward developing more adaptable, lightweight, and user-friendly BCI systems.

To address these limitations and distinguish our approach from existing subject-specific metaheuristic methods, the incremental contribution of this study is the development of a highly generalizable, fixed few-channel selection framework that integrates a Genetic Algorithm with Bayesian linear discriminant analysis. In this framework, GA provides a global combinatorial search to avoid local optima in channel selection, while BLDA serves as a robust fitness evaluator to mitigate the risk of overfitting on noisy EEG data. Instead of applying this process dynamically to recalibrate channels for each new user, we utilized this integrated GA-BLDA approach across a large cohort to statistically identify a universal few-channel configuration, aiming to significantly improve user experience and system practicality. The main contributions are as follows:

1. A universal few-channel selection framework: We integrated a genetic algorithm with Bayesian linear discriminant analysis to extract a fixed, cross-subject channel configuration. Unlike existing metaheuristic approaches that rely on subject-specific recalibration, this strategy avoids individualized algorithmic searches prior to operation, offering a plug-and-play solution that reduces calibration overhead while preserving core decoding performance.
2. Enhanced system practicality and user comfort: By transitioning to a minimal electrode layout, the proposed framework structurally minimizes equipment preparation and post-experiment cleaning. This simplified physical setup directly addresses the usability barriers of traditional multi-channel arrays, effectively lowering the mental and physical demands on users and providing a more viable option for prolonged BCI operation in practical scenarios.

In summary, this study not only provides a theoretical basis and practical schemes for the rapid and lightweight deployment of BCI systems, but also breaks the experience bottleneck of traditional multi-channel systems under the premise of maintaining high performance, and is expected to promote BCI technology to advance towards broader complex real-world scenarios.

## 2. Materials and Methods

### 2.1. Subject

In this study, a total of 48 healthy volunteers (12 females, 36 males, aged between 18 and 26) were recruited. All subjects participated in a comparative experiment based on two different channel selection schemes, aiming to explore the impact of the number of channels on the subjects. In addition, all subjects had normal or corrected-to-normal vision, no history of neurological or psychiatric diseases, and no previous experience in EEG-related experiments. This study was approved by the Ethics Committee of Guangzhou Maritime University. All subjects provided written informed consent and had a full understanding of the experimental purpose and procedure.

### 2.2. EEG Signal Acquisition and Preprocessing

**Figure 1:**
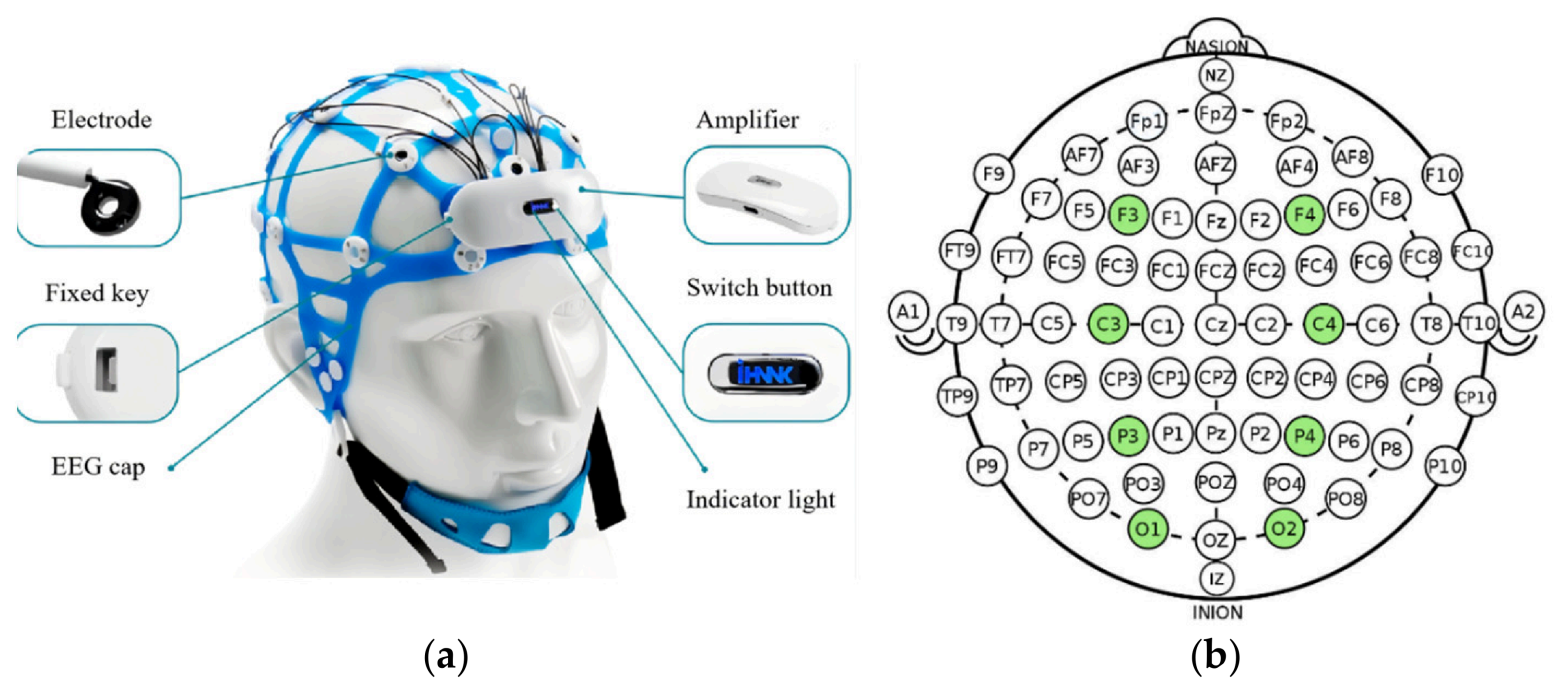
Overview of the wearable EEG cap: (**a**) wearable EEG cap; (**b**) EEG electrode placement. (The green circles in (**b**) indicate the 8 selected EEG channels used in this study).

In this study, a wearable EEG cap developed by South China Brain Control Technology Co., Ltd. (Guangzhou, China) was used for signal acquisition, as shown in Figure 1a. The device is wirelessly connected to the computer via Bluetooth 4.2 to record the EEG signals of the subjects in real time during the experimental tasks. This EEG cap is equipped with 8 electrodes: F3, F4, C3, C4, P3, P4, O1, and O2, as shown in Figure 1b. The electrode layout follows the international 10–20 system standard, effectively covering relevant brain regions such as the frontal lobe, central region, parietal lobe, and occipital lobe, with a signal sampling rate of 250 Hz. In this study, wet electrodes were used for EEG signal acquisition. Conductive paste was injected into the contact area between the electrodes and the scalp to effectively reduce impedance, ensuring that the contact impedance of all acquisition channels dropped below 10 kΩ, thereby guaranteeing the acquisition quality and high signal-to-noise ratio of the raw EEG signals.

Data preprocessing and feature extraction are important prerequisites for achieving high-precision target classification of EEG signals [24,25]. In this paper, for a single flash stimulus, data preprocessing is first performed: taking the time of stimulus onset as the zero point, the complete EEG data segment of [−200, 600] ms is extracted. Then, the average amplitude of the pre-stimulus 200 ms (i.e., [−200, 0] ms) signal is calculated and subtracted from the entire [−200, 600] ms data to complete baseline correction. Subsequently, a 4th-order infinite impulse response (IIR) band-pass filter is applied to all baseline-corrected data segments to retain the 0.1~25 Hz frequency band information, and a notch filter is applied to eliminate the 50 Hz power-line interference. Finally, the entire filtered signal is down-sampled by a factor of 5 to reduce data dimensionality and improve subsequent computational efficiency.

Then, feature extraction is performed: the data segment corresponding to [0, 600] ms of the stimulus after down-sampling is extracted as the analysis time window (150 sampling points at the original 250 Hz sampling rate, and 30 sampling points after down-sampling by a factor of 5). This time window is evenly divided into 4 sub-windows with a length of 150 ms, and the mean and variance of the EEG signals within each sub-window are respectively calculated as local time-domain features. The features of all time windows and the features of all selected channels are concatenated to form the feature vector of a single round of stimulation. Finally, to effectively eliminate spontaneous EEG background noise and highlight the P300 evoked potential, this study superimposes and averages the feature vectors extracted from 10 rounds of stimulation for the same target, thereby obtaining the final trial feature vector used for classification.

In the model training and evaluation phase, to ensure a fair comparison, a unified data partitioning strategy is adopted for the offline and online experiments of all subjects. The model uses 10-fold cross-validation, dividing the dataset by trial, strictly ensuring the independence and data balance of the training set and the test set. Specifically, in each iteration of the cross-validation process, the dataset is partitioned into a 9:1 ratio, utilizing 90% of the data for model training and the remaining 10% for testing.

### 2.3. The Design of Virtual Reality Scenario

**Figure 2:**
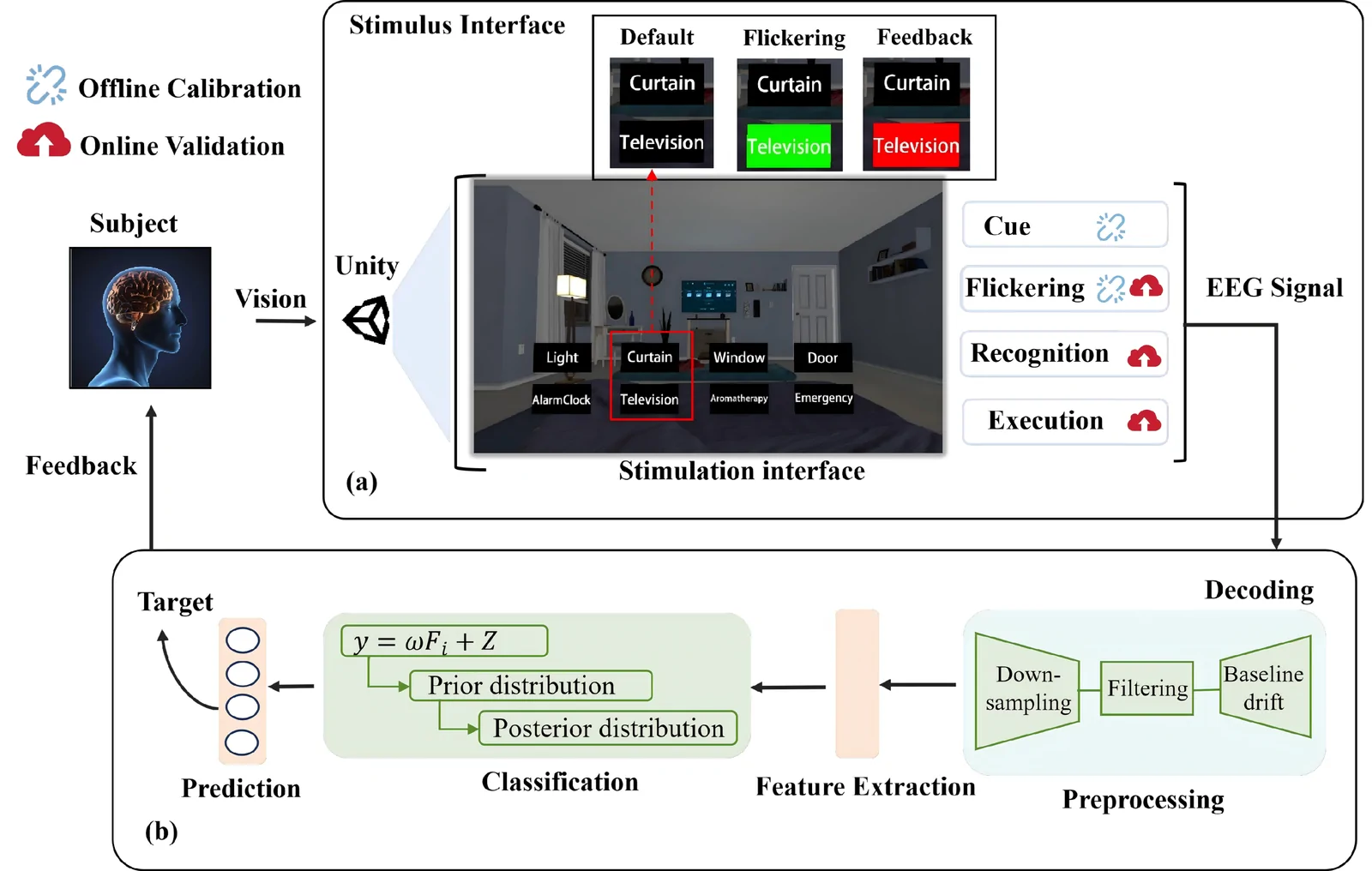
Overview of the system: (**a**) stimulus interface of the virtual reality scene; (**b**) signal decoding process.

To provide immersive and effective visual stimuli to evoke target EEG signals, this study developed a “room function control” virtual reality interaction scene based on the Unity3D engine (version 2022.3 LTS, Unity Technologies, San Francisco, CA, USA). The stimulus interface of the virtual reality scene is presented through a monitor with a resolution of 1920 × 1080 pixels and a refresh rate of 60 Hz, as shown in Figure 2a. The stimulus interface is designed with a first-person perspective, enabling subjects to naturally focus on multiple interactive objects within the virtual room (such as curtains, TV, doors, lights, etc.), with a virtual control button equipped below each object. To ensure the effectiveness of the visual evoked paradigm, the target button is designed with three dynamic response states: it appears black in the default state; the button turns red and lasts for 2 s in the task cue phase or system recognition feedback phase; in the core stimulation phase, the button flashes alternately in black and green, with each flash duration set to 0.1 s.

At the level of signal decoding and command feedback, the entire system constructs a closed-loop brain–computer interaction data flow. As shown in Figure 2b, the synchronously acquired EEG signals sequentially undergo preprocessing (including baseline correction, filtering, and down-sampling) and feature extraction; subsequently, the extracted feature vectors are fed into the classifier for probability prediction, and the specific label of the target button is output. Finally, the system transmits the predicted command back to Unity to realize the control of interactive objects.

### 2.4. Decoding Algorithm

Bayesian linear discriminant analysis (BLDA) is a powerful classification method that exhibits good classification performance across multiple trials [26]. This study adopts this classification algorithm to conduct target classification for the experiments. Assuming the regression target is Y, for each feature vector $F_{i}$ and noise z following a Gaussian distribution, the following relationship exists:

(1) $$Y=\omega^{T}F_{i}+z$$

Here, $\omega \in R^{s}$ represents the feature weight, which determines the importance of each feature in the regression target.

For a new input sample matrix F, the distribution function of its predicted value y can be obtained:

(2) p(y|β,α,F,S)=∫p(y|β,F,ω)p(ω|β,α,S) dω

where β is the inverse variance of the noise z, α is the prior variance of the weight ω, p(y|β,F,ω) is the likelihood function of the predicted value y, and p(ω|β,α,S) is the posterior distribution of the predicted value y.

The regression target value u corresponding to the predictive distribution function can be calculated by the following equation:

(3) $$u=\mu^{T}F$$

where the mean u is the regression target value, and μ is the mean of the posterior distribution of the predicted value y.

### 2.5. Channel Selection

**Figure 3:**
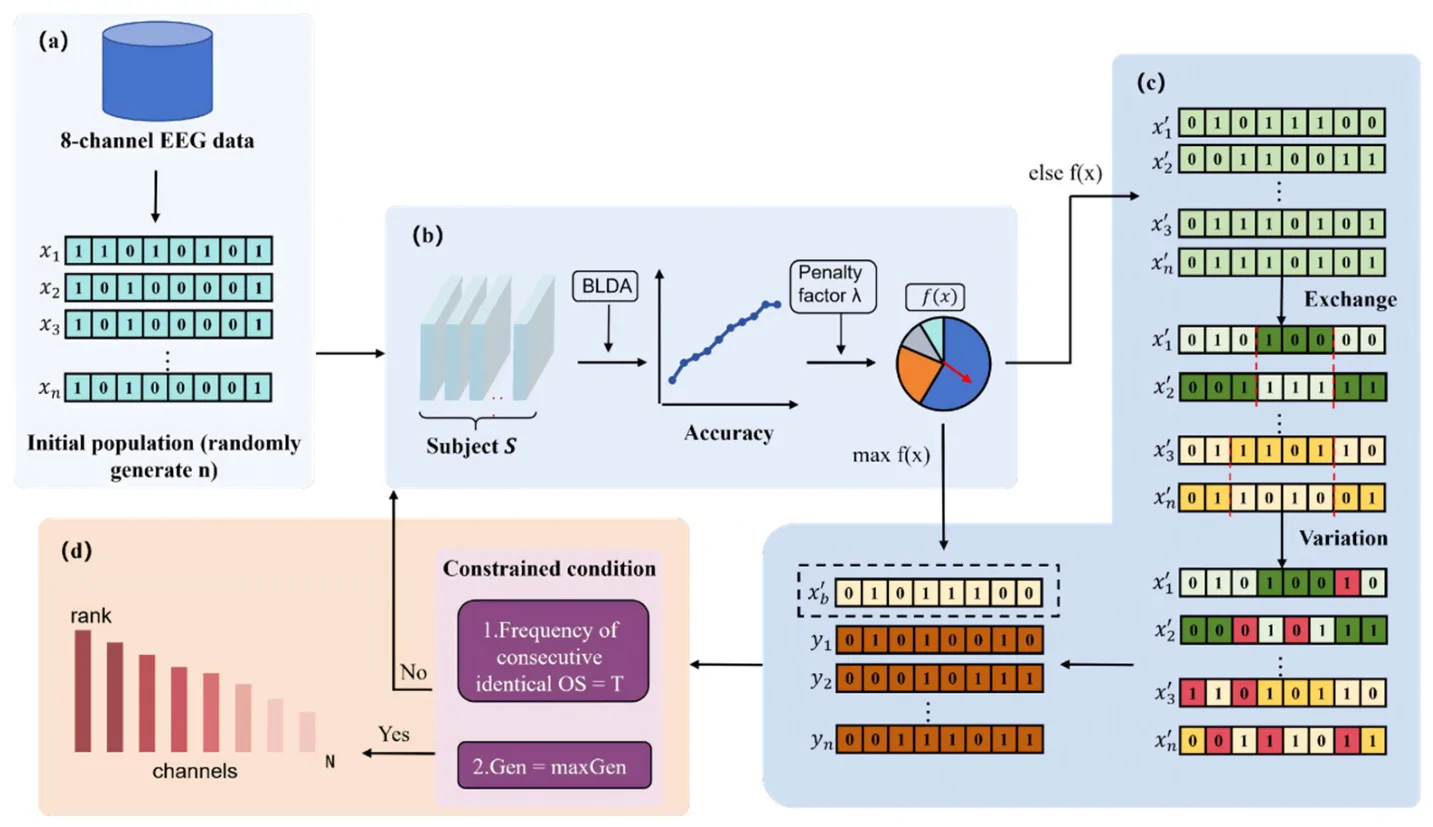
Flowchart of the GA and BLDA algorithms: (**a**) population initialization; (**b**) fitness evaluation; (**c**) genetic operation and population update; (**d**) constraint conditions and frequency sorting. The arrows indicate the execution flow of the algorithm. In part (**c**), different colors are utilized to illustrate the genetic operations: distinct color pairs (e.g., light and dark green for x′_1_ and x′_2_; light and dark yellow for x′_3_ and x′_n_) denote the chromosomal segments exchanged between individuals during the crossover process. Meanwhile, the red color highlights the specific genes that have undergone mutation (i.e., bits flipping from 1 to 0, or 0 to 1).

Reducing the number of channels improves the applicability and efficiency of brain–computer interface systems in practical deployment [27,28]. Therefore, this study proposes a channel selection strategy combining the genetic algorithm (GA) and Bayesian linear discriminant analysis (BLDA) to effectively reduce the number of channels while maintaining classification accuracy. The complete execution logic of this algorithm is shown in Figure 3, specifically covering four stages:

Population initialization. As shown in Figure 3a, for a single subject (8-channel EEG data), the algorithm first randomly generates an initial population consisting of n individuals. Individuals in the population are encoded as binary vectors $x_{i}=[x_{i1},x_{i2},\ldots ,x_{iN}]$ of length N=8, where $x_{ij}=1$ indicates that the j channel is selected, and 0 otherwise. The completely random initialization strategy introduces no single-subject priors. It has low computational overhead, effectively maintains population diversity, and reduces the risk of falling into local optima early, providing an unbiased starting point for the global search [29].

Fitness evaluation. As shown in Figure 3b, in the individual optimization loop for subject S, the system extracts the channel data corresponding to the individual to train the BLDA classifier and obtains the classification accuracy. To strike a balance between classification performance and a minimal number of channels, a single-objective weighted fitness function is constructed to calculate the final score (corresponding to the f(x) pie chart in Figure 3b):

(4) $$f\left(x\right)=Acc\left(x\right)-\lambda \frac{{\left\|x\right\|}_{1}}{N}$$

where Acc(x) is the accuracy of the current channel combination obtained through 10-fold cross-validation with the BLDA classifier; λ is the weight factor controlling the strength of the channel penalty; and ${\left\|x\right\|}_{1}$ is the actual number of channels selected by the individual. The algorithm evaluates the performance of each individual using f(x), which directly determines the individual’s retention probability in subsequent genetic operations.

Genetic operation and population update. As shown in Figure 3c, the population generates two divergent paths based on fitness scores to update the offspring: the first is elitism, where the individual with the highest current fitness (max f(x)) directly bypasses genetic operations and is completely retained into the new population (denoted as $x_{b}^{\prime }$), ensuring that the optimal solution is not destroyed; the second, for the remaining individuals in the population (else f(x)), sequentially executes Exchange and Variation operations to generate a completely new sequence of offspring individuals $\left(y_{1},{\;y}_{2},\ldots ,{\;y}_{n}\right)$. The above two parts are combined to form the new generation population, completing the intergenerational update of the solution space while strictly maintaining the population size unchanged.

Constraint conditions and frequency sorting. As shown in Figure 3d, the newly generated population must undergo a constrained condition judgment. The iteration process triggers the termination command (the “Yes” path) when either of the following conditions is satisfied: first, the number of consecutive times the current optimal solution remains unchanged reaches a preset value T (Frequency of consecutive identical OS = T); second, the current number of iterations reaches the maximum threshold (Gen = max Gen). If the above conditions are not met, the process re-enters the loop (the “No” path). In the offline data processing stage, once the independent optimization for all subjects is completed, the optimal channel combinations obtained from everyone are aggregated. The occurrence frequency of each channel is then counted and sorted in descending order, thereby yielding the final rank results.

Randomization and Reproducibility of the GA Framework. Because the Genetic Algorithm relies on stochastic processes, it inherently introduces randomness into the optimization pipeline. In our channel selection framework, this randomness specifically occurs during the generation of the initial binary population in Stage (a), the data partitioning for the 10-fold cross-validation in Stage (b), the selection of crossover points, and the probability-driven bit-flipping during mutation in Stage (c). To control for this variability and guarantee exact reproducibility, we standardized the pseudo-random number generators across our software environment. Specifically, we assigned a fixed global random seed in Python before initiating any offline data processing. Fixing the seed ensures that the algorithm’s evolutionary trajectory, intergenerational updates, and final channel rankings in Stage (d) are fully deterministic. Consequently, any subsequent execution of the framework using our offline dataset and parameter configurations will perfectly replicate the universal 3-channel subset identified in this study.

In summary, during the channel selection stage, the algorithm first processes each subject’s data independently. By executing the closed-loop iterations from (a) to (d), it obtains the optimal channel subset matching the EEG characteristics of each subject. Subsequently, through frequency counting and descending sorting (Rank) of the optimal subsets from all subjects, the top three channels with the highest occurrence frequencies are extracted as the universal channel combination, providing the optimal channel configuration for subsequent experiments.

### 2.6. The Design of Experimental Procedures

Prior to the formal experiment, 8-channel offline data were first collected from the 48 recruited subjects. The optimal fewer-channel combination was selected using the channel selection strategy shown in Figure 3, which is collectively referred to as the “fewer-channel scheme” hereafter.

**Figure 4:**
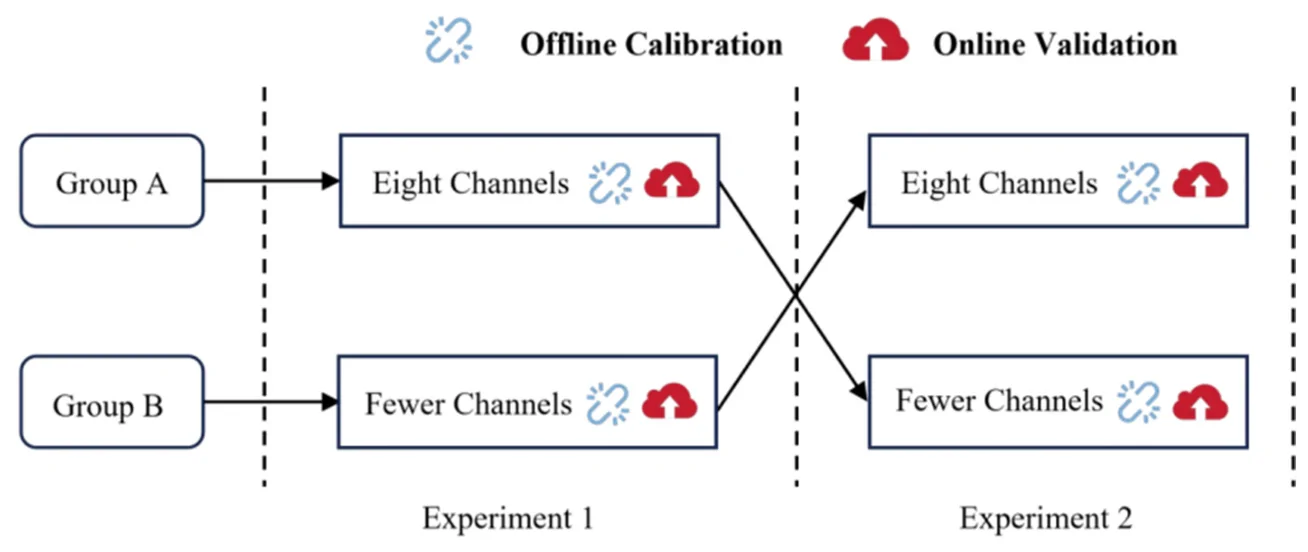
Experimental workflow and system validation design: During the AB/BA crossover experiment phase for evaluating system performance, Groups A and B sequentially alternated between the eight-channel scheme and the fewer-channel scheme. Each experiment includes offline calibration and online validation sessions for the current system, with a 48 h interval between the two experiments to eliminate fatigue and learning effects.

Two weeks later, we conducted a formal comparative experimental study between the 8-channel and fewer-channel schemes. To ensure experimental standardization and eliminate the interference of learning effects, we designed an AB/BA crossover experiment, as shown in Figure 4. The 48 subjects were randomly and equally divided into Group A (*n* = 24) and Group B (*n* = 24). In Experiment 1, Group A initially used the traditional 8-channel scheme; whereas, Group B used the fewer-channel scheme. Following a 48 h washout period to eliminate fatigue and muscle memory, Experiment 2 took place, during which the two groups swapped the system schemes they used. This crossover design strictly balances the order of system usage and the volume of training data, thereby maximizing the reliability of the performance comparison results.

**Figure 5:**
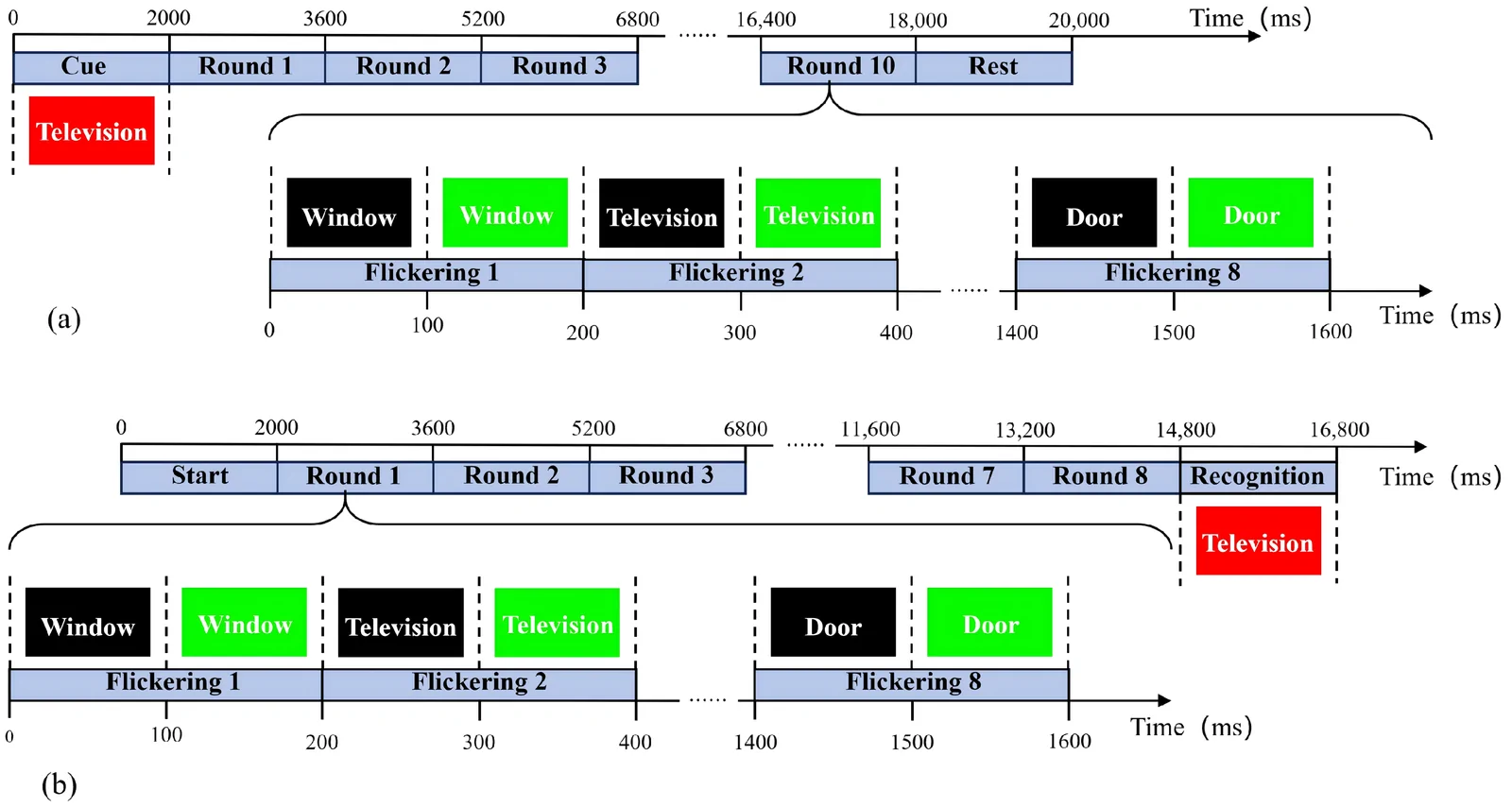
Illustrations of the offline and online experimental paradigms: (**a**) offline paradigm; (**b**) online paradigm.

As shown in Figure 5a, the offline calibration experiment is based on the classic Oddball paradigm. Each subject was required to consecutively complete 20 trials, with each trial lasting a total of 20 s. The first 2 s of a trial served as the cue phase, during which the target button was highlighted in red to remind the subject to concentrate and fixate on the target. This was followed by a 16 s stimulus presentation phase comprising 10 consecutive rounds of stimulation. In each round (lasting 1.6 s), the 8 buttons in the 2 × 4 matrix (as shown in Figure 2a) flashed individually in a pseudo-random order. A single flash cycle lasted 0.2 s (consisting of a 0.1 s black background and a 0.1 s green highlight). Subjects were required to silently count when the target button turned green and to keep their gaze steady when non-target buttons flashed. A 2 s rest period was provided at the end of the trial to alleviate visual fatigue. It should be noted that the offline calibration settings for the pre-experiment and the formal experiment were identical. The offline data from the pre-experiment were used for channel selection via the strategy shown in Figure 3; whereas, the offline data from the formal experiment were utilized to train the model using the BLDA classification algorithm for the subsequent online experiments.

As shown in Figure 5b, the paradigm of the online validation experiment was similar to that of the offline phase. The difference was that the total duration for a single target recognition was shortened to 16.8 s, and the stimulus phase was reduced from 10 rounds in the offline stage to 8 rounds. After all 8 rounds of stimulation concluded, the system utilized the pre-trained classification model to decode the real-time extracted EEG signals. During the feedback phase at the end of the trial, the predicted target button was highlighted in red for 2 s, and the corresponding functional command in the virtual scene was simultaneously executed. Throughout the entire online testing process, subjects were required to continuously perform the visual fixation and counting tasks to comprehensively evaluate the real-time control performance of the BCI system in practical application scenarios.

### 2.7. Data Analysis Methods

#### 2.7.1. Performance Evaluation

Classification accuracy (Acc) and information transfer rate (ITR) are critical performance metrics for evaluating BCI systems [30]. In this study, the offline experiment assesses the performance of the BCI system using Acc and ITR. The specific definitions are as follows:

(5) $$Acc=\frac{TP+TN}{TP+TN+FP+FN}$$

where TP, TN, FP, and FN represent the numbers of true positive, false positive, true negative, and false negative samples, respectively;

(6) $$ITR=60\left[\log_{2}M+Acc\;\log_{2}Acc+\left(1-Acc\right)\log_{2}\frac{1-Acc}{M-1}\right]/RT$$

where M is the total number of commands, and RT is the response time of the system.

#### 2.7.2. Workload Evaluation Methods

The NASA-TLX is a multidimensional method for assessing workload [31]. Upon completion of the entire experiment, the subjects were also required to complete the NASA-TLX questionnaire to quantify the burden imposed by the 8-channel and fewer-channel schemes. The specific evaluation dimensions are presented in Table 1.

## 3. Results

### 3.1. Experimental Environment and Parameter Settings

All offline data processing and online real-time decoding tasks in this study were conducted on a standard desktop workstation. The hardware environment consisted of an Intel Core i7-11700K CPU @ 3.60 GHz, 32 GB of RAM, and a Windows 10 64-bit operating system. The EEG signal preprocessing, genetic algorithm (GA) channel selection, and BLDA classification models were implemented using Python 3.9. The virtual reality interactive scenes were developed and rendered using the Unity3D engine. Regarding the core parameter settings, the raw EEG signals were uniformly band-pass filtered at 0.1–25 Hz and down-sampled by a factor of 5. For the GA-based channel selection, the optimal parameters utilized across all evaluations were derived from our orthogonal experiments: a population size of 30, maximum iterations of 10, a mutation rate of 0.07, and an elite retention rate of 0.05.

### 3.2. Channel Selection Analysis

**Figure 6:**
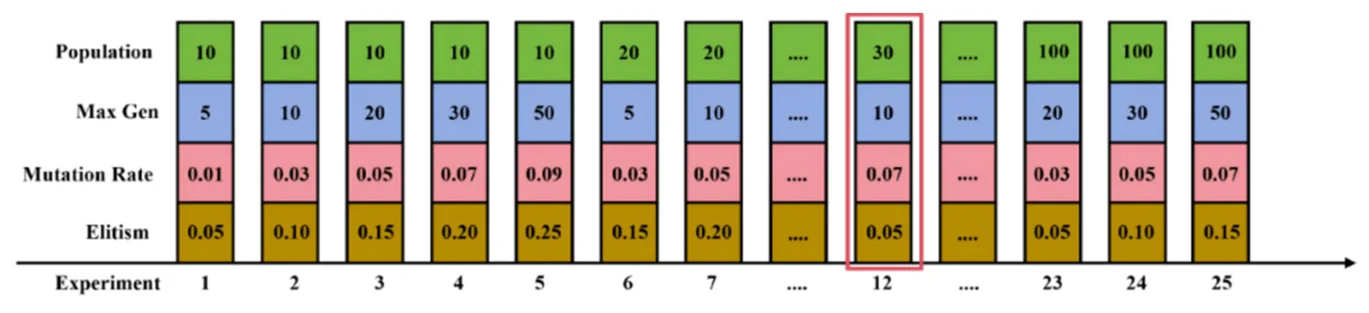
Genetic algorithm parameter design based on orthogonal experiment (four factors, five levels, 25 combinations). The red box highlights the optimal parameter combination.

The population size, number of iterations, mutation rate, and elite retention rate of the genetic algorithm typically require multiple rounds of parameter tuning, a process that entails a substantial workload. Orthogonal experimental design is an effective method for addressing the time-consuming nature of multi-factor and multi-level experiments. For the offline data from the pre-experiment in this study, we designed the population size (10, 20, 30, 50, 100), number of iterations (5, 10, 20, 30, 50), mutation rate (0.01, 0.03, 0.05, 0.07, 0.09), and elite retention rate (0.05, 0.1, 0.15, 0.2, 0.25). Based on the $L_{25}(5^{4})$ orthogonal array, 25 parameter combinations were established for selection, as shown in Figure 6.

Here, for each parameter combination, the channel ranking for each subject can be determined using our channel selection scheme (Figure 3). Based on these rankings, we obtained the selection frequencies of different channels across all subjects in the pre-experiment. As shown in Table 2, the frequency of C3 is 1, indicating that C3 is the first ranked channel for all 48 subjects; meanwhile, the frequency of C4 is 0.95, meaning that 46 subjects selected C3 and C4 as their two-channel combination. Furthermore, Table 2 displays the selection frequency of each channel across all subjects under the parameter combination of Orthogonal Experiment 12 from Figure 6. Sorted in descending order of frequency, the channels are: C3, C4, P4, P3, F3, F4, O2, and O1.

**Figure 7:**
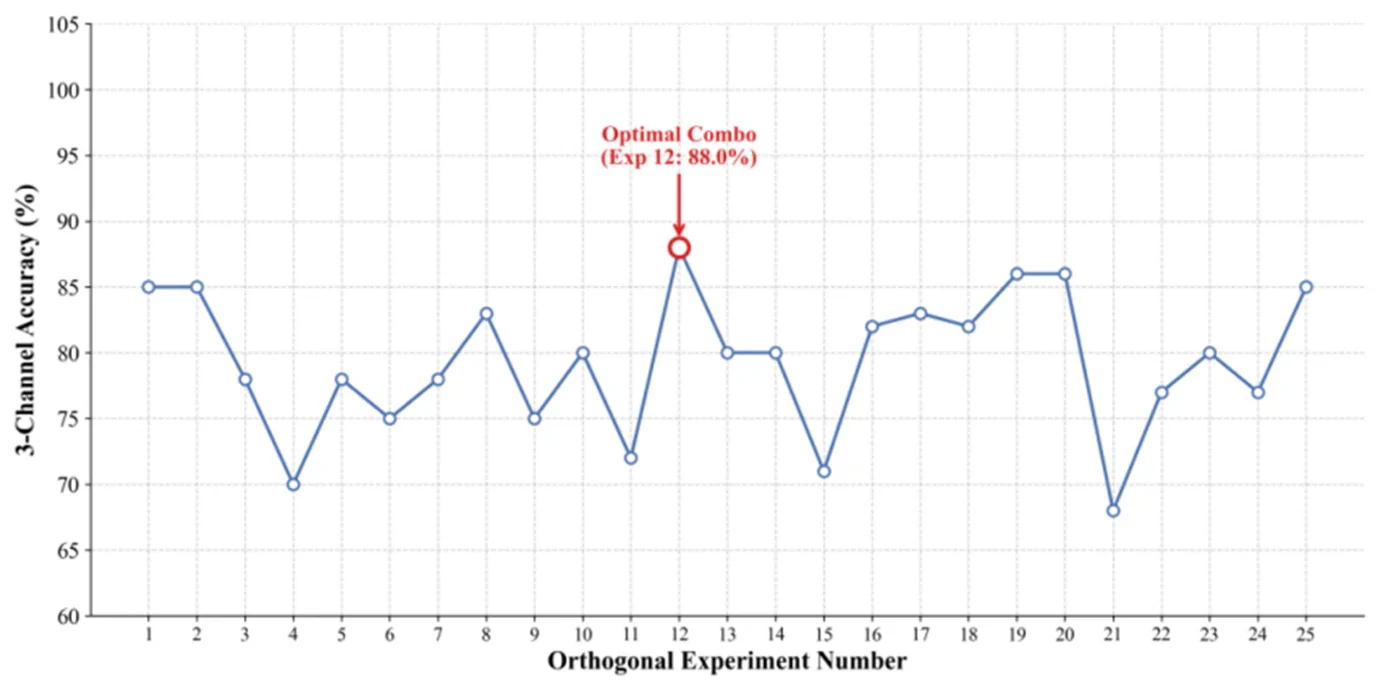
Three-channel classification accuracy across the 25 orthogonal experiments. The parameter combination in Experiment 12 achieved the highest accuracy and was adopted for all subsequent evaluations.

**Figure 8:**
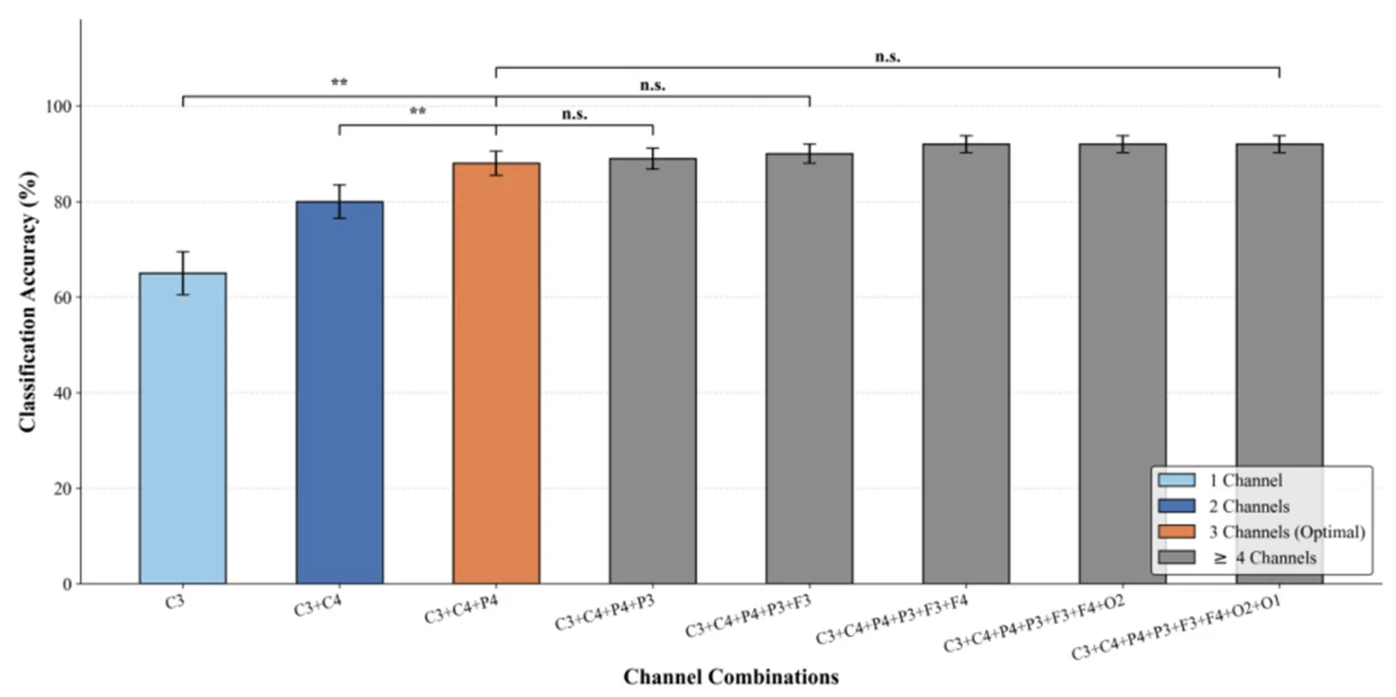
Accuracy under different channel combinations in Orthogonal Experiment 12. “n.s.” and “**” indicate the significance levels of the paired-sample t-test between the fewer-channel and eight-channel schemes, representing p > 0.05 and p < 0.01, respectively.

Figure 7 presents the three-channel classification accuracy across the 25 orthogonal experiments. The results demonstrate that the parameter combination evaluated in Experiment 12 outperformed all other configurations, achieving the highest accuracy of 88.0%. Consequently, this specific parameter set was adopted as the standard genetic algorithm configuration for all subsequent offline and online experiments. Furthermore, as shown in Figure 8, when utilizing the genetic parameters established in Orthogonal Experiment 12, the average accuracy of the three-channel configuration (88.0%) was significantly higher (*p* < 0.01, paired-sample *t*-test) than that of the one-channel (65.0%) and two-channel (80.0%) setups. However, increasing the array to four channels (89.0%) or more (90.0–92.0%) yielded no significant improvement in average accuracy (*p* > 0.05, paired-sample *t*-test). This statistical plateau confirms that the three-channel layout (C3, C4, P4) provides an optimal balance between performance and electrode reduction, thoroughly validating its selection for the proposed fewer-channel scheme.

### 3.3. Offline Calibration Experimental Results

**Figure 9:**
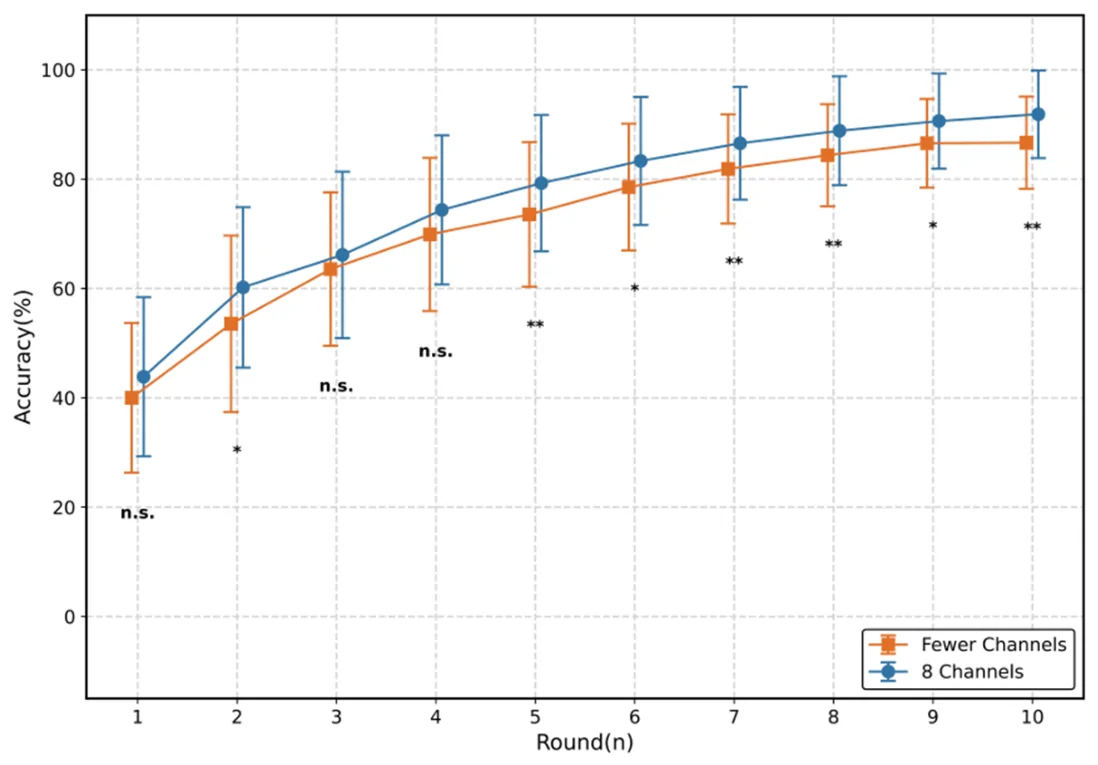
Classification accuracy: The orange line represents the classification accuracy of the fewer-channel (three-channel) scheme, and the blue line represents that of the eight-channel scheme. “n.s.”, “*”, and “**” indicate the significance levels of the paired-sample t-test between the fewer-channel and eight-channel schemes, representing p > 0.05, p < 0.05, and p < 0.01, respectively.

Figure 9 details the offline classification accuracy of the two-channel configurations under continuous detection (rounds 1 to 10). Overall, as the number of flashing rounds increased, the classification accuracies of both schemes exhibited a similar upward trend. Specifically, the average accuracy of the traditional eight-channel scheme gradually increased from 43.85% in round 1, reaching a peak of 91.88% in round 10; similarly, the average accuracy of the fewer-channel (three-channel) scheme gradually rose from 40.00% in round 1 to a peak of 86.67% in round 10. As shown in the figure, the classification performance of both channel schemes in round 10 was notably higher than that in rounds 1 to 5. Furthermore, in rounds 7 to 10, the accuracy of the traditional eight-channel scheme was significantly higher than that of the fewer-channel scheme (all p < 0.05, paired-sample t-test). Despite the statistically significant difference, the absolute performance gap between the two was controlled within a very narrow margin: in the final 10th round, the accuracies of the two groups differed by only about 5.2% (91.88% vs. 86.67%).

**Figure 10:**
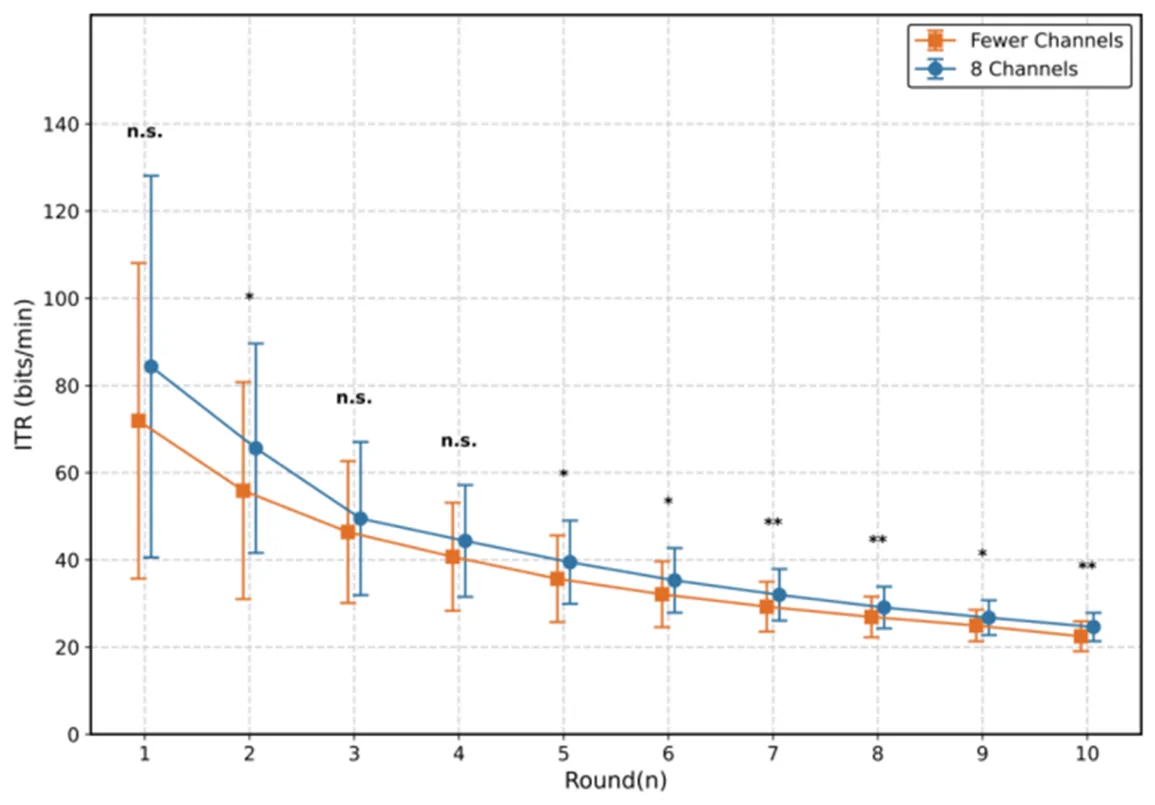
Information transfer rate: The orange line represents the information transfer rate of the fewer-channel (three-channel) scheme, and the blue line represents that of the eight-channel scheme. “n.s.”, “*”, and “**” indicate the significance levels of the paired-sample t-test between the fewer-channel and eight-channel schemes, representing p > 0.05, p < 0.05, and p < 0.01, respectively.

Figure 10 illustrates in detail the information transfer rate (ITR) for the two channel setups across continuous detection (rounds 1 to 10). Generally, with an increasing number of flash rounds, both schemes demonstrated a marked downward trend in ITR. Specifically, the traditional eight-channel scheme’s average ITR peaked at 84.33 bits/min in the first round before steadily declining to 24.60 bits/min by the 10th round. Likewise, the fewer-channel (three-channel) scheme’s average ITR achieved its maximum of 71.91 bits/min in round 1 and dropped to 22.47 bits/min in round 10. The figure indicates that the ITR for both configurations in round 1 was substantially higher than in rounds 5 through 10. Additionally, from rounds 7 to 10, the ITR of the eight-channel scheme significantly outperformed the fewer-channel scheme (all p < 0.05, paired t-test). Even with this statistical significance, the absolute performance discrepancy was kept extremely small: at the final 10th round, the ITR gap between the two groups was merely 2.13 bits/min (24.60 bits/min vs. 22.47 bits/min).

Overall, although the traditional eight-channel configuration maintains a slight performance advantage at the statistical level, the proposed fewer-channel (three-channel) scheme still provides robust and highly competitive performance for offline P300 classification while significantly reducing the number of electrodes.

### 3.4. Online Verification of Experimental Results

As shown in Table 3, the average online accuracy of the fewer-channel scheme was 90.21% ± 5.36%, with an ITR of 23.66 ± 4.02 bits/min; whereas, the online accuracy of the eight-channel scheme was 92.40% ± 5.55%, with an ITR of 27.52 ± 3.76 bits/min. Although the fewer-channel scheme was slightly lower than the eight-channel scheme in terms of information transfer rate, the difference in classification accuracy between the two did not reach statistical significance (p > 0.05, paired-sample t-test). Furthermore, under the fewer-channel scheme, certain subjects (S26, S39, S41, S44, and S45) achieved 100% accuracy, and 10 subjects (S6, S7, S16, S19, S22, S27, S31, S32, S44, and S45) outperformed their own results from the eight-channel scheme. These results demonstrate that the proposed fewer-channel scheme possesses high online operational performance, enabling the rapid execution of tasks in real-world scenarios.

### 3.5. Workload Evaluation

**Figure 11:**
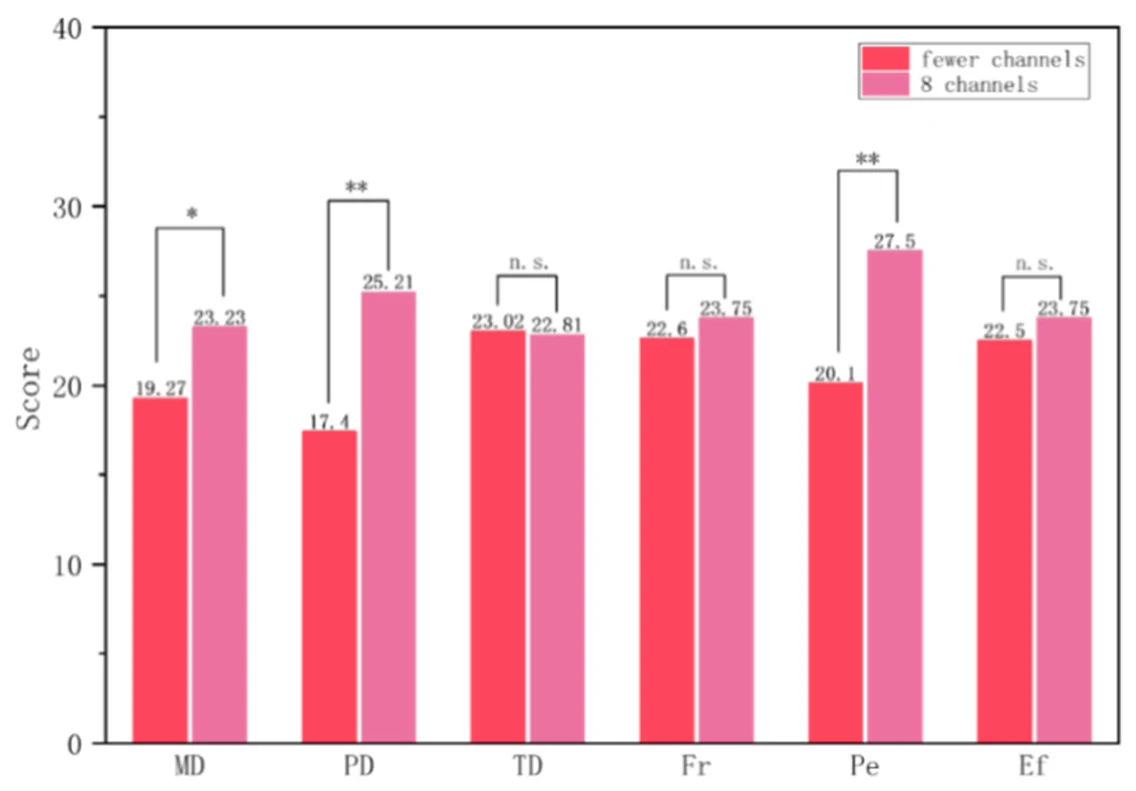
Workload assessment results for the fewer-channel and eight-channel schemes based on the NASA-TLX questionnaire. MD, PD, TD, Fr, Pe, and Ef stand for mental demand, physical demand, temporal demand, frustration, performance, and effort, respectively. “n.s.”, “*”, and “**” indicate the significance levels of the paired-sample t-test between the fewer-channel and eight-channel schemes, corresponding to p > 0.05, p < 0.05, and p < 0.01, respectively.

Upon completion of the experiment, each subject was required to complete the NASA-TLX questionnaire to assess the workload associated with the two schemes (the eight-channel and fewer-channel schemes). As illustrated in Figure 11, the workload score for the proposed fewer-channel scheme was significantly lower than that of the traditional eight-channel scheme. The fewer-channel group scored significantly better than the eight-channel group in the dimensions of mental demand (19.27 ± 7.72 vs. 23.23 ± 8.09, p < 0.05, paired-sample t-test) and physical demand (17.40 ± 7.72 vs. 25.21 ± 7.78, p < 0.01, paired-sample t-test). Additionally, the performance score of the fewer-channel group was also significantly better than that of the eight-channel group (20.10 ± 7.96 vs. 27.50 ± 8.12, p < 0.01, paired-sample t-test). These findings indicate that reducing the number of electrode channels can effectively alleviate the subjects’ cognitive and operational burdens, lowering their anxiety regarding their own performance while maintaining the quality of task execution. Notably, although there were no significant differences (p > 0.05) between the fewer-channel and eight-channel groups in temporal demand (23.02 ± 6.82 vs. 22.81 ± 7.43), frustration level (22.60 ± 8.05 vs. 23.75 ± 8.60), and effort (22.50 ± 8.93 vs. 23.75 ± 9.02), the scores of the fewer-channel group were generally lower than those of the eight-channel group. This suggests that the fewer-channel BCI system may possess greater superiority for long-term use.

## 4. Discussion

To reduce the number of channels and enhance practicality, a fixed fewer-channel optimization strategy based on a genetic algorithm (GA) is proposed. Under the premise of maintaining decoding accuracy without significant loss, we effectively reduced the number of channels from 8 to 3. First, we systematically optimized the GA parameters via an orthogonal experimental design (*L*_25_(5^4^)) based on the pre-experiment, thereby identifying GA parameters more suitable for the BCI system (population size: 30, iteration count: 10, mutation rate: 0.07, elitism ratio: 0.05). Next, using the genetic algorithm (Figure 3), we selected the top three channels that appeared most frequently among the 48 subjects: C3, C4, and P4. These channels, located in the frontoparietal and parietal regions, achieved frequencies of 1.00, 0.95, and 0.90, respectively. This result not only validates the physiological plausibility of the channel selection (which is consistent with the typical neurophysiological characteristics of the P300 component) but also statistically demonstrates the robustness of this channel combination across a large sample size.

The accuracy results of the offline experiment (Figure 9) indicate that the fewer-channel system optimized by the genetic algorithm (GA) achieved an average accuracy of 86.67% among the 48 subjects. Although there was a significant difference compared to the eight-channel system (91.88%), the gap between the two was controlled within a very narrow margin, differing by only about 5.2%. This result is comparable to the manual channel selection method based on expert experience proposed by Krusienski et al. (accuracy: 92.5%) [9]. On the other hand, the ITR experimental results (Figure 10) demonstrate that despite the fewer-channel BCI system (22.47 ± 4.02 bits/min) being slightly lower than the eight-channel BCI system (24.60 ± 3.76 bits/min), the information transfer rates of the two groups differed by a mere 2.13 bits/min (24.60 bits/min vs. 22.47 bits/min).

Regarding the online experimental results, the proposed fewer-channel BCI system demonstrated favorable real-time performance and robustness in practical interactive scenarios. The average accuracy of the fewer-channel group was 90.21% ± 5.36%, with an ITR of 23.66 ± 4.02 bits/min. Although these values were slightly lower than those of the eight-channel group (Accuracy: 92.40% ± 5.55%, ITR: 27.52 ± 3.76 bits/min), no significant difference was observed (p > 0.05). These results indicate that the proposed fewer-channel system and the eight-channel system perform comparably in terms of online performance. Considering that the fixed-round approach can avoid the computational complexity of the BLDA classifier caused by dynamic round adjustments, we adopted a fixed eight-round stimulus superposition method in the online system. Compared with adaptive round optimization methods, the fixed-round approach may prolong the single-command time to some extent, but it can achieve more stable and higher decoding performance [10].

To further highlight the advantages of the proposed GA-BLDA framework, Table 4 compares our study with several recent representative few-channel BCI studies. For instance, the wearable P300 system utilizing a four-channel configuration (3 EEG + 1 EOG) developed by Hu et al. [32], which achieved an impressive accuracy of 94.03%, relies on a hybrid modality, specifically blink detection, to maintain high performance. In the motor imagery (MI) domain, the multi-task transfer learning method on an eight-channel system employed by Long et al. [33], which achieved an accuracy of 78.01%, reduces the calibration burden without utilizing hybrid signals. More recently, the advanced sparse Bayesian modeling for dynamic P300 channel selection applied by Ma et al. [34], which achieved exceptional precision, still requires computationally intensive, subject-specific adaptive recalculations during operation. Particularly, when compared to the approach presented by Ma et al., the primary novelty of our work lies in the paradigm shift from subject-specific dynamic adaptation to a universal fixed framework. While Ma et al. rely on individualized channel searches (typically retaining around five electrodes) during the calibration phase for every new user, our proposed framework offloads the computational burden entirely to prior offline statistical analysis. By deriving a fixed, universal three-channel layout, our system strictly minimizes the electrode count from 5 to 3 and completely eliminates the pre-use algorithmic recalculation cost. This zero-calibration-cost approach fundamentally enhances the plug-and-play capability and practical deployment efficiency of the BCI system.

Similarly, while heuristic and evolutionary algorithms have significantly advanced channel selection, existing studies using these methods typically adopt an application strategy that strictly relies on subject-specific optimization due to high inter-subject variability. For example, Esfahani et al. [35] applied the SPEA-II algorithm for motor imagery BCIs, and Martínez-Cagigal et al. [13] explored diverse meta-heuristics (e.g., DFGA, NSGA-II) for P300 BCIs. Both approaches require running computationally intensive wrapper algorithms to find a custom channel layout specifically for each new user. Consequently, their optimal subsets remain subject-specific and typically retain a relatively higher number of electrodes.

In contrast, although our study is also based on a heuristic algorithm, we introduce a fundamentally different application strategy. Instead of dynamically executing the genetic algorithm for each user prior to operation, we utilized the GA-BLDA framework to evaluate offline data across a large cohort, statistically deriving a fixed, universal three-channel combination (C3, C4, P4) that relies entirely on pure EEG signals. It requires neither auxiliary modalities (such as EOG) nor subject-specific algorithmic searches before use. By achieving a highly competitive online accuracy of 90.21% using merely three electrodes, our framework significantly reduces equipment preparation time and maximizes user comfort, thereby offering a highly practical, plug-and-play solution for real-world BCI deployment.

**Table 4:** Comparison of the proposed method with recent representative few-channel BCI studies.

Study	BCI Paradigm	Channel Selection	Final Channels	Accuracy
Hu et al. [32]	P300 + EOG (Hybrid)	Empirical layout	4 (3 EEG + 1 EOG)	94.03%
Long et al. [33]	Motor Imagery	Multi-task Transfer Learning	8	78.01%
Ma et al. [34]	P300	Adaptive Sparse Bayesian Modeling	Adaptive	~100% (Median)
Esfahani et al. [35]	Motor Imagery	SPEA-II + Regularized CSP	11–19	~80.00%
Martínez-Cagigal et al. [13]	P300	DFGA	7–20	84.60–100%
**Our Study**	P300	GA + BLDA	3	86.67% (Offline) 90.21% (Online)

The bold values indicate the best overall performance.

To rigorously validate our system’s efficiency, we re-implemented three representative P300 channel selection strategies from recent literature and evaluated them under identical conditions using offline cross-validation on our 48-subject dataset. As summarized in Table 5, the DFGA meta-heuristic [13] and the Adaptive Sparse Bayesian model [34] yielded average offline accuracies of 87.50% and 88.10%, respectively. These methods, however, rely on computationally demanding, subject-specific channel searches that typically require 5 to 8 electrodes. A paired-sample *t*-test specifically evaluating offline accuracy revealed no significant differences (*p* > 0.05) between these complex methods and our fixed three-channel framework (86.67%). Furthermore, our universally optimized, pure-EEG layout achieved a significantly higher offline accuracy (*p* < 0.05) than the four-channel hybrid configuration (3 EEG + 1 EOG) adapted from Hu et al. [32]. This direct offline comparison confirms the practical superiority of the proposed GA-BLDA framework: it delivers decoding performance comparable to complex, dynamic multi-channel algorithms while strictly minimizing the electrode count and eliminating pre-use computational calibration.

The workload assessment results based on the NASA-TLX indicate that the system can provide subjects with a good user experience. As shown in Figure 11, the average scores across all six dimensions for both the eight-channel and fewer-channel groups were below 30, suggesting that controlling the virtual reality scenes under either configuration does not induce a high workload. Regarding specific dimension comparisons, the scores for mental demand (p < 0.05), physical demand (p < 0.01), and performance (p < 0.01) in the fewer-channel group were significantly lower than those in the eight-channel group. This demonstrates that the subjects obtained a more relaxed and comfortable experience with the fewer-channel scheme. This improvement in experience is directly attributed to the simplified physical setup. The fewer-channel scheme (requiring only three electrodes) drastically reduced the equipment preparation time for subjects from 30 min to 3 min, and decreased the subsequent cleaning time by 80%. Furthermore, this corroborates the conclusions of previous studies that cumbersome electrode installation leads to a decline in user experience [36,37]. At the same time, there were no significant differences between the fewer-channel and eight-channel groups in the scores for temporal demand, frustration, and effort (all p > 0.05), indicating that reducing the number of channels did not increase the operational difficulty for the subjects. Furthermore, addressing the visual fatigue potentially induced by prolonged fixation on the virtual interface, the P300 stimulus interface designed in this study effectively circumvented the risk of dizziness by simplifying the interaction logic and reducing visual complexity (Figure 2). None of the subjects reported discomfort after the experiment. Overall, the fewer-channel selection scheme proposed in this study not only greatly liberates users during the preparation and cleaning phases, but also significantly reduces their physical and mental workload while ensuring interaction performance, providing an important reference for the practical application of BCI systems in complex scenarios in the future.

Although this study has validated the efficacy of the fewer-channel BCI system in virtual reality control tasks, several inherent challenges and limitations remain within the proposed framework. First, from a technical perspective, drastically reducing the electrode array to merely three channels inherently sacrifices spatial resolution and signal redundancy. This poses a fundamental challenge, as it can make the system potentially more susceptible to environmental noise and motion artifacts compared to high-density multi-channel arrays. Although our proposed GA-BLDA framework demonstrated robust resistance to these issues in the current controlled experiments, mitigating severe noise interference in highly complex, real-world mobile scenarios remain a critical challenge for future few-channel BCIs. Second, the participant cohort was restricted to healthy young adults, excluding patients with motor impairments or elderly populations; thus, the clinical efficacy requires further validation. Third, although the fixed channel combination (C3, C4, and P4) demonstrated stable performance, a strictly universal layout faces the inherent challenge of not fully accounting for individual differences in EEG signals across all diverse users. Adopting a personalized channel strategy might yield higher system performance. Furthermore, this study utilized wet electrodes. Future research needs to further verify the robustness of this fewer-channel scheme in dry electrode systems without conductive gel, in order to enhance its convenience for daily application. Building upon these findings, future research can be advanced in the following directions: First, developing dynamic channel optimization algorithms to balance universality and personalized demands via real-time feedback. Second, introducing deep learning models to compensate for the information loss caused by channel reduction, thereby enhancing classification accuracy. Third, exploring the system’s stability in complex environments (e.g., multi-task interference and mobile scenarios) to bolster its potential for practical application. In summary, this study demonstrates the rationality of the fewer-channel selection scheme through both theoretical and experimental validation. This not only provides a scientific basis for channel selection in BCI applications for rehabilitation medicine and intelligent assistive devices, but also offers a novel approach to improving the overall user experience.

## 5. Conclusions

Addressing the pain point of cumbersome multi-channel deployment in current P300-BCI systems, this study proposes a fewer-channel (C3, C4, and P4) selection scheme based on a heuristic algorithm, with the core objective of enhancing user experience and system practicality. Experiments conducted on 48 subjects demonstrated that the proposed scheme maintained a high level of performance comparable to the traditional eight-channel system in terms of both offline and online accuracy and information transfer rate (e.g., online accuracy: 90.21% vs. 92.40%), with no significant performance disadvantage observed. Furthermore, the workload assessment conclusively confirmed that, while ensuring interactive efficacy, the fewer-channel configuration significantly alleviates the mental and physical burdens on the users. This fully demonstrates that streamlining the channels not only effectively breaks the physical constraints of traditional BCI systems but also delivers a more relaxed and comfortable real-world user experience. In the future, we will explore the generalization ability of this lightweight scheme in dry electrode systems, clinical populations, and complex scenarios, while further optimizing the algorithm by integrating deep learning. Overall, this study confirms the feasibility of achieving “high performance driven by fewer channels,” providing crucial theoretical and practical support for advancing highly practical and user-friendly BCI systems toward real-world implementation.

## Figures and Tables

**Table 1:** Qualitative evaluation of the NASA-TLX.

Item	Description	Rating Scale
Mental Demand	How much mental activity (thinking, decision-making, calculation, memory, searching, etc.) is required to complete the task? From a mental aspect, is the task easy or difficult, simple or complex for you?	Low (0) to High (100)
Physical Demand	How much physical effort (pushing, pulling, turning, controlling, moving, etc.) is required to complete the task? From a physical aspect, is the task easy or difficult for you?	Low (0) to High (100)
Temporal Demand	Is the pace of completing the task slow or fast for you? Do you feel at ease or flustered?	Low (0) to High (100)
Frustration	During the process of completing the task, how small or large is the degree of frustration or annoyance you feel?	Low (0) to High (100)
Effort	How much effort do you put into completing this task, small or large?	Low (0) to High (100)
Performance	How is the achievement in achieving the task objectives? How satisfied are you with the achieved performance?	Low (0) to High (100)

**Table 2:** Channel selection frequencies based on the genetic algorithm (population size: 30, iteration count: 10, mutation rate: 0.07, elitism ratio: 0.05).

Passage	C3	C4	P4	P3	F3	F4	O2	O1
Frequency	1.00	0.95	0.90	0.80	0.50	0.40	0.20	0.10

Note: Frequency here denotes the unitless selection proportion across all 48 subjects.

**Table 3:** Accuracy and information transfer rate of the 48 subjects under the eight-channel and fewer-channel (three-channel) schemes.

Subject	Fewer Channels		8 Channels	
	Acc (%)	ITR (Bits/min)	Acc (%)	ITR (Bits/min)
1	95.00	27.84	95.00	28.71
2	80.00	19.59	85.00	22.41
3	85.00	18.00	85.00	23.08
4	80.00	17.39	85.00	22.88
5	90.00	17.60	90.00	25.12
6	95.00	22.92	80.00	18.93
7	90.00	19.41	85.00	22.04
8	90.00	25.32	95.00	29.22
9	90.00	18.13	90.00	26.82
10	85.00	22.13	95.00	28.76
11	85.00	22.96	100.00	32.44
12	80.00	17.34	80.00	21.38
13	85.00	22.01	95.00	29.35
14	85.00	21.24	90.00	26.81
15	85.00	21.50	90.00	26.07
16	90.00	22.36	80.00	20.58
17	85.00	22.57	90.00	23.99
18	85.00	23.36	95.00	28.57
19	95.00	27.56	90.00	26.05
20	90.00	17.25	90.00	25.06
21	85.00	21.42	85.00	21.63
22	90.00	12.92	85.00	22.26
23	90.00	21.77	95.00	28.42
24	95.00	23.58	95.00	28.65
25	85.00	22.13	90.00	25.72
26	100.00	30.03	100.00	31.11
27	95.00	23.25	90.00	24.95
28	90.00	25.55	95.00	28.92
29	90.00	24.99	100.00	32.83
30	95.00	27.77	100.00	33.61
31	95.00	20.50	90.00	26.99
32	95.00	30.88	90.00	26.27
33	90.00	24.93	90.00	25.79
34	95.00	28.60	100.00	34.02
35	85.00	23.42	95.00	28.88
36	95.00	27.34	100.00	32.46
37	85.00	22.38	95.00	29.73
38	90.00	24.25	95.00	30.58
39	100.00	29.35	100.00	33.72
40	90.00	27.32	95.00	30.13
41	100.00	29.37	100.00	33.56
42	90.00	25.68	95.00	28.56
43	90.00	26.98	95.00	28.91
44	100.00	29.56	95.00	30.00
45	100.00	27.37	95.00	28.64
46	90.00	26.45	95.00	28.96
47	90.00	26.22	95.00	27.61
48	90.00	25.00	95.00	29.93
Average ± SD	90.21 ± 5.36	23.66 ± 4.02	92.40 ± 5.55	27.52 ± 3.76

**Table 5:** Offline performance comparison of representative P300 algorithms evaluated under the identical simulation environment (our 48-subject dataset).

Study	BCI Paradigm	Final Channels	Offline Accuracy (%)	Offline ITR (Bits/min)	*p*-Value
Hu et al. [32]	P300 + EOG	4	84.30 ± 6.50	20.15 ± 4.80	0.038
Ma et al. [34]	P300	5	87.50 ± 5.80	23.10 ± 4.20	0.285
Martínez-Cagigal et al. [13]	P300	7	88.10 ± 5.40	23.50 ± 4.00	0.164
**Our Study**	P300	3	86.67 ± 6.25	22.47 ± 4.02	Ref.

The bold values indicate the best overall performance. Note: The evaluations were conducted using offline cross-validation on the 48-subject dataset. The presented *p*-values are specifically for the offline accuracy, calculated via a paired-sample *t*-test comparing each existing method against our proposed method.

## Data Availability

The data presented in this study are available on request from the corresponding author.

## References

[1] Yuan J., Zhang R., Zhao Y., Zhou W., Tian L., Liu G. (2026). PG-MCTFormer: A Prior-Guided Multi-Scale Convolutional Transformer for Interpretable Motor Imagery EEG Classification. Biomimetics.

[2] Kim S., Lee S., Kang H., Kim S., Ahn M. (2021). P300 Brain–Computer Interface-Based Drone Control in Virtual and Augmented Reality. Sensors.

[3] Khan S., Kallis L., Mee H., El Hadwe S., Barone D., Hutchinson P., Kolias A. (2025). Invasive Brain–Computer Interface for Communication: A Scoping Review. Brain Sci..

[4] Hu H., Wang Z., Zhao X., Li R., Li A., Si Y., Wang J., Zhou T., Xu T. (2025). A Survey on Brain-Computer Interface-Inspired Communications: Opportunities and Challenges. IEEE Commun. Surv. Tutor..

[5] Cattan G.H., Andreev A., Mendoza C., Congedo M. (2019). A comparison of mobile VR display running on an ordinary smartphone with standard PC display for P300-BCI stimulus presentation. IEEE Trans. Games.

[6] Wang H., Guo H., Zhang K., Gao L., Zheng J. (2022). Automatic sleep staging method of EEG signal based on transfer learning and fusion network. Neurocomputing.

[7] Pitt K.M., Cole Z.J., Zosky J. (2024). Applying functional animation to pictorial symbols for supporting P300–brain–computer interface access to augmentative and alternative communication devices by children. Int. J. Hum.–Comput. Interact..

[8] Prabhakar S.K., Won D.O. (2026). A Consolidated Framework for the Detection of Alzheimer’s Disease Using EEG Signals and Hybrid Models. Biomimetics.

[9] Krusienski D.J., Sellers E.W., McFarland D.J., Vaughan T.M., Wolpaw J.R. (2008). Toward enhanced P300 speller performance. J. Neurosci. Methods.

[10] Qu J., Wang F., Xia Z., Yu T., Xiao J., Yu Z., Gu Z., Li Y. (2018). A Novel Three-Dimensional P300 Speller Based on Stereo Visual Stimuli. IEEE Trans. Hum.-Mach. Syst..

[11] Ai J., Meng J., Mai X., Zhu X. (2023). BCI Control of a Robotic Arm Based on SSVEP With Moving Stimuli for Reach and Grasp Tasks. IEEE J. Biomed. Health Inf..

[12] McCrimmon C.M., Fu J.L., Wang M., Lopes L.S., Wang P.T., Karimi-Bidhendi A., Liu C.Y., Heydari P., Nenadic Z., Do A.H. (2017). Performance Assessment of a Custom, Portable, and Low-Cost Brain-Computer Interface Platform. IEEE Trans. Biomed. Eng..

[13] Martínez-Cagigal V., Santamaría-Vázquez E., Hornero R. (2022). Brain–computer interface channel selection optimization using meta-heuristics and evolutionary algorithms. Appl. Soft Comput..

[14] Bhandari V., Londhe N.D., Kshirsagar G.B. (2024). A Systematic Review of Computational Intelligence Techniques for Channel Selection in P300-Based Brain Computer Interface Speller. Artif. Intell. Appl..

[15] Chen W., Chen S.-K., Liu Y.-H., Chen Y.-J., Chen C.-S. (2022). An Electric Wheelchair Manipulating System Using SSVEP-Based BCI System. Biosensors.

[16] Luo J., Zhu M., Xiao Y., Long Y., Zhang X., Cao H., Atai J., Xiao J., Chen X. (2026). An Immersive P300 Brain–Computer Interface Based on 3D Morphological Stimuli and Self-Adaptive Bayesian Linear Discriminant Analysis. Biomimetics.

[17] Nijboer F., Sellers E.W., Mellinger J., Jordan M.A., Matuz T., Furdea A., Halder S., Mochty U., Krusienski D.J., Vaughan T.M. (2008). A P300-based brain–computer interface for people with amyotrophic lateral sclerosis. Clin. Neurophysiol..

[18] González J., Ortega J., Damas M., Martín-Smith P., Gan J.Q. (2019). A new multi-objective wrapper method for feature selection—Accuracy and stability analysis for BCI. Neurocomputing.

[19] Ponmalar A., Vijayakumar K., Lakshmipriya C., Karthikeyan M., Gracelin Sheena B., Beslin Pajila P., Siva Subramanian R. (2024). Meta-heuristics and machine learning applications in complex systems. Metaheuristic and Machine Learning Optimization Strategies for Complex Systems.

[20] Rao Z., Lu Z., Xiao J., Li K., Zhu M., Guan Z., Chen Y., Cao H., Li Y. (2026). Calibration-Free Online Detection in Wearable Motor Imagery Brain-Computer Interfaces. IEEE Trans. Neural. Syst. Rehabil. Eng..

[21] Gkintoni E., Halkiopoulos C. (2025). Mapping EEG Metrics to Human Affective and Cognitive Models: An Interdisciplinary Scoping Review from a Cognitive Neuroscience Perspective. Biomimetics.

[22] Mounesi Rad S., Danishvar S. (2024). Emotion Recognition Using EEG Signals through the Design of a Dry Electrode Based on the Combination of Type 2 Fuzzy Sets and Deep Convolutional Graph Networks. Biomimetics.

[23] Attallah O. (2024). ADHD-AID: Aiding Tool for Detecting Children’s Attention Deficit Hyperactivity Disorder via EEG-Based Multi-Resolution Analysis and Feature Selection. Biomimetics.

[24] Martínez-Cagigal V., Hornero R. (2017). Selección de Canales en Sistemas BCI basados en Potenciales P300 mediante Inteligencia de Enjambre. Rev. Iberoam. Autom. Inform. Ind. RIAI.

[25] Hong K.S., Khan M.J., Hong M.J. (2018). Feature Extraction and Classification Methods for Hybrid fNIRS-EEG Brain-Computer Interfaces. Front. Hum. Neurosci..

[26] Delgado J.M.C., Achanccaray D., Villota E.R., Chevallier S. (2020). Riemann-Based Algorithms Assessment for Single- and Multiple-Trial P300 Classification in Non-Optimal Environments. IEEE Trans. Neural. Syst. Rehabil. Eng..

[27] Feess D., Krell M.M., Metzen J.H. (2013). Comparison of sensor selection mechanisms for an ERP-based brain-computer interface. PLoS ONE.

[28] Handiru V.S., Prasad V.A. (2016). Optimized Bi-Objective EEG Channel Selection and Cross-Subject Generalization With Brain–Computer Interfaces. IEEE Trans. Hum.-Mach. Syst..

[29] Atum Y., Pacheco M., Acevedo R., Tabernig C., Biurrun Manresa J. (2019). A comparison of subject-dependent and subject-independent channel selection strategies for single-trial P300 brain computer interfaces. Med. Biol. Eng. Comput..

[30] McFarland D.J., Sarnacki W.A., Wolpaw J.R. (2003). Brain-computer interface (BCI) operation: Optimizing information transfer rates. Biol. Psychol..

[31] Kosch T., Karolus J., Zagermann J., Reiterer H., Schmidt A., Woźniak P.W. (2023). A Survey on Measuring Cognitive Workload in Human-Computer Interaction. ACM Comput. Surv..

[32] Hu L., Zhu J., Chen S., Zhou Y., Song Z., Li Y. (2024). A Wearable Asynchronous Brain-Computer Interface Based on EEG-EOG Signals with Fewer Channels. IEEE Trans. Biomed. Eng..

[33] Long T., Wan M., Jian W., Dai H., Nie W., Xu J. (2023). Application of multi-task transfer learning: The combination of EA and optimized subband regularized CSP to classification of 8-channel EEG signals with small dataset. Front. Hum. Neurosci..

[34] Ma G., Zhong Y., Li M., Nie Y., Kang J. (2026). Sparse Bayesian Modeling of EEG Channel Interactions Improves P300 Brain-Computer Interface Performance. arXiv.

[35] Esfahani M.M., Sadati H., Calhoun V.D. (2024). Optimizing Brain-Computer Interface Performance: Advancing EEG Signals Channel Selection Through Regularized CSP and SPEA II Multi-Objective Optimization. arXiv.

[36] Rao Z., Zhu J., Lu Z., Zhang R., Li K., Guan Z., Li Y. (2024). A Wearable Brain-Computer Interface with Fewer EEG Channels for Online Motor Imagery Detection. IEEE Trans. Neural Syst. Rehabil. Eng..

[37] Xing X., Wang Y., Pei W., Guo X., Liu Z., Wang F., Ming G., Zhao H., Gui Q., Chen H. (2018). A High-Speed SSVEP-Based BCI Using Dry EEG Electrodes. Sci. Rep..

